# Reliable renewable energy forecasting for climate change mitigation

**DOI:** 10.7717/peerj-cs.2067

**Published:** 2024-05-23

**Authors:** Walid Atwa, Abdulwahab Ali Almazroi, Nasir Ayub

**Affiliations:** 1 College of Computing and Information Technology at Khulais, Department of Information Technology, University of Jeddah, Jeddah, Saudi Arabia; 2 Department of Creative Technologies, Air University, Islamabad, Pakistan

**Keywords:** Renewable energy, Hybrid AI model, Forecasting, Deep learning, Climate change

## Abstract

Accurate prediction of electricity generation from diverse renewable energy sources (RES) plays a pivotal role in optimizing power schedules within RES, contributing to the collective effort to combat climate change. While prior research often focused on individual energy sources in isolation, neglecting intricate interactions among multiple sources, this limitation frequently leads to inaccurate estimations of total power generation. In this study, we introduce a hybrid architecture designed to address these challenges, incorporating advanced artificial intelligence (AI) techniques. The hybrid model seamlessly integrates a gated recurrent unit (GRU) and a ResNext model, and it is tuned with the modified jaya algorithm (MJA) to capture localized correlations among different energy sources. Leveraging its nonlinear time-series properties, the model integrates meteorological conditions and specific energy source data. Additionally, principal component analysis (PCA) is employed to extract linear time-series data characteristics for each energy source. Application of the proposed AI-infused approach to a renewable energy system demonstrates its effectiveness and feasibility in the context of climate change mitigation. Results reveal the superior accuracy of the hybrid framework compared to more complex models such as decision trees and ResNet. Specifically, our proposed method achieved remarkable performance, boasting the lowest error rates with a normalized RMSE of 6.51 and a normalized MAPE of 4.34 for solar photovoltaic (PV), highlighting its exceptional precision in terms of mean absolute errors. A detailed sensitivity analysis is carried out to evaluate the influence of every element in the hybrid framework, emphasizing the importance of energy correlation patterns. Comparative assessments underscore the increased accuracy and stability of the suggested AI-infused framework when compared to other methods.

## Introduction

The need for energy has grown dramatically to meet everyday human needs and activities in tandem with the social economy’s fast expansion (Semieniuk et al., 2021). Power generation has increased significantly as a result of the rising reliance on energy. Traditional fossil fuels have triggered significant environmental harm, contributing to issues like air pollution and global warming (Hassan et al., 2021). The release of greenhouse gases from these fuels intensifies climate change. To counteract these challenges, there is a growing emphasis on transitioning to RESs, driven by the advancement of green technologies and the enactment of stringent “zero carbon emission” regulations (Ebhota & Tabakov, 2021). This shift towards green development has become integral to people’s lives. Many nations are actively investing in the development of RESs, utilizing sustainable energy sources to generate electricity.

As per the International Energy Agency (IEA) (Kober et al., 2020), it is projected that RESs will represent approximately 70% of all new power production capacity by 2040. This underscores a global commitment to transitioning away from conventional energy sources towards more sustainable and environmentally friendly alternatives. The smart microgrid idea has led to a substantial expansion in the application of RESs due to their numerous benefits, which include minimum environmental pollution, security of the energy supply, and the realization of sustainable development. RESs can be utilized to provide all or part of a region’s power demands; they typically comprise of loads, energy generation, and energy storage components. For both individual producers and system operators in RESs, estimating the quantity of electricity produced from renewable sources is essential.

As an illustration, power plants must submit transmission deals to the australian national electricity market up to forty hours beforehand, with the option to amend the offers up to five minutes before to the dispatch (Simshauser, 2021). Additionally, precise forecasting of electricity generation from RESs holds the potential to enhance the efficiency of power dispatch, optimize the scheduling of power resources, and contribute to increased economic gains for energy-related enterprises. This, in turn, facilitates improved operational coordination within power systems, ensuring the security and reliability of energy supplies.

Biogas, biofuel (Rahman, Farrok & Haque, 2022), geothermal energy, small-scale hydroelectricity, solar photovoltaic, thermal solar, wind, and solar thermal are examples of RESs that may be used in RESs. To improve the multi-energy generation prediction’s accuracy, it is important to investigate how multiple energy sources coordinate (Ang et al., 2022). The weather has a significant impact on energy generation forecast, particularly for wind and solar power, which puts the smart microgrid’s electricity scheduling at risk. Electricity may be produced from solar radiation using PV modules or concentrated solar thermal power and wind energy from wind farms. Solar thermal power can only be produced by direct normal irradiance, whereas PV electricity can be produced by worldwide horizontal irradiance (Law et al., 2014). With several wind sources, an increasing number of wind farms have been established, particularly in America, China, and Germany (Sahu, Hiloidhari & Baruah, 2013).

The variability in weather conditions contributes to the unpredictability of both wind and solar power generation, establishing a spatial–temporal correlation between them. Accurate solar and wind energy forecasting reduces the risk of blackouts or even outages in the system and decreases the price of energy balancing, which is the basis for RESs’ capacity to anticipate the supply of power (Tu et al., 2021). This article studies the power generation projection in a RES while accounting for the local correlations of numerous sources of renewable energy in order to reduce uncertainty in the electricity supply.

### Motivation

The essential need to strengthen the stability and dependability of RESs, particularly in the context of growing environmental concerns and emergency scenarios, is the basis behind this study. A revolutionary transition to sustainable energy resources is required in order to address climate change and reduce our dependence on traditional fossil fuels (Kabeyi & Olanrewaju, 2022). With the ability to decrease the effects of climate change and guarantee a sustainable energy future, RESs, provide a promising path forward for clean and green energy (Al-Shetwi, 2022). Robust and adaptableRESs are even more crucial in emergency scenarios, such as emergencies or disruptions to traditional energy networks. Precise forecasting of renewable energy output is essential for daily operations as well as for quickly and efficiently handling unanticipated events (Alkabbani et al., 2021). This study aims to combine green and nature-inspired approaches into renewable energy forecasting, driven by the critical need to develop resilient energy systems that can resist disasters and aid in disaster recovery efforts.

### Contribution

By addressing important issues and presenting novel approaches, our study significantly advances the area of renewable energy forecasting. The following are this work’s primary technical contributions:

1. Hybrid architecture for projecting renewable energy: We suggest a unique hybrid design to capture intricate interactions between various RESs, combining ResNext (Pant, Yadav & Gaur, 2020) and GRU (Wang, Liao & Chang, 2018) models. In contrast to other methods that frequently concentrate on estimating distinct energy sources independently, our hybrid model takes into account the localized correlations between many sources. Because of the complex interrelationships between various energy components, this design yields more precise estimates of overall power generation.
2. Implementing PCA for feature extraction: We use PCA for feature extraction in order to increase the dataset’s temporal dimension and interpretability. Effective feature selection is aided by PCA, which decreases the dimensionality of the data while keeping important information. Improved model performance is made possible by this contribution, which makes it possible to express linear time-series features more effectively.
3. Rigorous performance evaluation standards: Using rigorous measures such as R-squared, explained variance, mean absolute error (MAPE), root mean squared error (RMSE), and RMS logarithmic error, we conduct a thorough performance review. This comprehensive examination guarantees a detailed appraisal of the model’s precision and accuracy, enabling a trustworthy comparison with the most recent versions. A more complex understanding of the model’s predicting ability is made possible by the use of several measures.
4. Superior predictive performance for hourly energy generation: Our proposed ResNeXt-GRU-MJA model outshines existing state-of-the-art methods by demonstrating a remarkable 15% improvement in hourly forecasting accuracy. This superior performance is substantiated through normalized measures of RMSE, MAPE, and MAE specifically tailored for hourly predictions. The model’s effectiveness in capturing short-term energy variations positions it as a valuable tool for applications requiring precise hourly forecasting.

### Article organization

The article is structured into several sections. ‘Related Work’ provides a systematic review of existing methods developed by researchers. In ‘System Model’, we present the innovative approach proposed to enhance the current work. ‘Simulation and Results’ delves into the experimental simulation, offering insights into the practical implementation of the methodology. The conclusion is then presented to summarize key findings and contributions.

## Related Work

In recent research efforts, a multitude of studies have been conducted to predict renewable energy output. However, a predominant trend has emerged, with a majority of these studies concentrating on individual energy sources, such as solar or wind power. Many researchers have developed large-scale solar power facilities since solar energy is one of the most promising sources. Some research projected solar irradiance instead of electricity generation because they used pricey solar irradiance meters by examining the interaction relationship between solar irradiance and the sky picture. Huang et al. (2021) introduced a solar irradiance forecasting approach aimed at enhancing the precision of solar power predictions. Likewise, Ghimire et al. (2023) presented a hybrid predictive model incorporating a multilayer perceptron (MLP) model and a convolution-based neural network. This model utilizes sky images to forecast global irradiation 15 min in advance. Matrenin et al. (2023) described a pipeline for a one-day ahead-of-time forecast of solar radiation and heat. It is based on four data-driven prediction steps and the imputation of past data. Michael et al. (2022) created a unique multivariate hybrid deep neural model that takes climatic effect into account when estimating sun irradiance one hour in advance. Real data from four different nations was used to validate the model. Alkhayat, Hasan & Mehmood (2022) carried out a comprehensive and in-depth investigation of the machine learning-based solar power prediction methods in order to address the inadequacies of the existing machine learning models and boost prediction accuracy.

Statistical and machine learning methods, such as decision trees (Mahmud et al., 2021), random forest (Liu & Sun, 2019), and time series ensembles (AlKandari & Ahmad, 2020), are employed for PV power forecasting as a result of the significant advancements in big data technology and measuring meters that store enormous amounts of data. To address the shortcomings of conventional AI modeling techniques, deep learning-based approaches significantly increase the forecast accuracy of PV power (Wang, Qi & Liu, 2019). The original PV power series was divided into four distinct neural network networks and sub-series using a hybrid deep learning model created by Khan, Walker & Zeiler (2022) that was based on wavelet packet decomposition. In the work presented by Gu et al. (2021), an innovative model is introduced, specifically designed for forecasting day-ahead PV power. This model incorporates principles dependent on time and integrates deep learning modeling into a framework for partially daily forecasting trends, serving as a guide for parameter modifications. The investigation conducted by Ahmed et al. (2020) involved a thorough examination and assessment of various modern forecasting techniques.

This comprehensive analysis encompassed considerations such as the forecast horizon, temporal aspects including date and time, scrutiny of input connections, preprocessing and postprocessing of data, optimization of network parameters, classification of weather patterns, estimation of uncertainties, and evaluations of overall efficacy. A few studies took into consideration the solar production of more than one location. In an earlier study, AlSkaif et al. (2020) presented an improved LSTM network for multi-region solar power output prediction that was optimized using particle swarm optimization. This strategy was applied to a genuine geographic region in Asia, demonstrating its efficacy. In a similar vein, another study (Nourani et al., 2022) examined the climatic and geographical details of multi-region solar production. In order to create an ideal ensemble prediction model, taking into account a variety of candidate characteristics, this study used automated machine learning. A genetic algorithm was used to identify the most appropriate parameters for the predictive model, which contributed to enhancing the feature selection process.

Despite the significance of solar thermal energy as a vital component of solar energy, there has been limited attention devoted to its forecasting in comparison to solar radiance or PV power output prediction in the existing body of research. The majority of studies have predominantly concentrated on predicting solar radiance levels or forecasting the output of PV systems, leaving solar thermal energy forecasting relatively understudied. Using a hybrid method based on deep learning and mechanism modeling for solar thermal prediction, Hu et al. (2021) developed a way to connect the spatial–temporal aspects between meteorological parameters and identify the key meteorological components. Dewangan, Singh & Chakrabarti (2020) used an instance of a solar Fresnel generator to anticipate the solar heat output 24 h ahead of time by combining a climatic model with a solar plant’s performance model.

Numerous studies have proposed effective strategies for forecasting wind speed and wind power, aiming to enhance the coordination of wind energy systems with power networks (ShobanaDevi et al., 2021). In the realm of wind energy forecasting, advancements have led to the categorization of forecasting models into two main types: deterministic prediction and uncertainty analysis. Deterministic prediction models can be broadly classified into physical and AI hybrid approaches. The physical approach, exemplified by numerical weather prediction (NWP), involves solving hydrodynamic and thermodynamic equations using computational methods (Patel et al., 2022). While physical models exhibit superior performance in long-term forecasting, they tend to underperform in short-term predictions and often require significant computational resources. On the other hand, statistical models, a subset of deterministic prediction, rely on mathematical theories such as the kalman filter (KF), copula theory, and Bayesian multiple kernel regression (Dong et al., 2022). These models leverage statistical knowledge to make predictions and are known for their versatility in handling various forecasting scenarios.

Statistical approaches are commonly used for time series forecasting, but they work best for linear data or data that has simple relationships between variables. When dealing with highly nonlinear data, their effectiveness reduces because they assume linearity. Although some statistical methods can be adjusted to accommodate nonlinear relationships to some extent, they may not perform as well as more advanced techniques designed specifically for nonlinear data, such as machine learning algorithms. Therefore, while statistical techniques can be used for nonlinear data, they may not provide the same level of accuracy or effectiveness as methods explicitly created for handling nonlinear relationships (Almazroi & Ayub, 2021).

Advanced learning machines and ANNs (Jiang et al., 2021) are two cutting-edge techniques that now surpass all others in the prediction of wind power and speed. In addition to the individual models, there has been a surge in the development of hybrid forecasting frameworks that outperform single models by amalgamating the strengths of multiple approaches. Notably, techniques such as decomposition and feature selection are specifically tailored for handling wind energy series (Almazroi & Ayub, 2023). Ensemble learning, on the other hand, takes a stride towards constructing a more robust model by incorporating numerous predictors. This trend underscores the pursuit of enhanced reliability and performance through the integration of diverse forecasting strategies. The related work is summarized in Table 1.

**Table 1:** Overview of related work on renewable energy forecasting.

**Ref**	**Problem addressed**	**Methodology employed**	**Achievements**	**Limitations/drawbacks**
Huang et al. (2021)	Enhanced precision of solar power predictions through solar irradiance forecasting	Solar irradiance forecasting approach	Improved accuracy in solar power predictions	Dependence on expensive solar irradiance meters
Ghimire et al. (2023)	Short-term forecasting of global irradiation using sky images	Hybrid predictive model with MLP and convolution- based neural network	Forecast global irradiation 15 min in advance	Limited applicability to short-term predictions
Matrenin et al. (2023)	One-day ahead forecast of solar radiation and heat	Four data-driven prediction steps and past data imputation	Accurate forecast of solar radiation and heat	Requirement of extensive past data
Michael et al. (2022)	Sun irradiance prediction considering climatic effects	Multivariate hybrid deep neural model	Improved accuracy in sun irradiance estimation	Necessity for real data from diverse nations
Alkhayat, Hasan & Mehmood (2022)	In-depth investigation of machine learning-based solar power prediction methods	Comprehensive analysis of existing models	Addressing inadequacies and improving prediction accuracy	No specific achievement highlighted
Mahmud et al. (2021); Liu & Sun (2019); AlKandari & Ahmad (2020)	PV power forecasting using statistical and machine learning methods	Decision trees, random forest, and time series ensembles	Advancements in big data technology	Challenges in handling highly nonlinear data
Wang, Qi & Liu (2019); Khan, Walker & Zeiler (2022)	Hybrid deep learning model for PV power forecasting	Division of original PV power series into distinct neural networks	Increased forecast accuracy of PV power	Complexity in model architecture
Gu et al. (2021)	Day-ahead PV power forecasting	Model with dependent- on-time principles and deep learning modeling	Partial daily forecasting of trends framework	Limited discussion on specific achievements
Ahmed et al. (2020)	Examination and assessment of diverse forecasting techniques	Thorough analysis covering various aspects	Comprehensive understanding of forecasting techniques	No specific achievement mentioned
AlSkaif et al. (2020)	Improved LSTM network for multi-region solar power output prediction	Optimization using particle swarm optimization	Demonstrated efficacy in a specific geographic region	Limited to a particular geographic context
Nourani et al. (2022)	Climatic and geographical details of multi-region solar production	Automated machine learning for creating an ensemble prediction model	Enhanced feature selection process	Challenges in utilizing automated machine learning
Hu et al. (2021)	Solar thermal prediction using a hybrid method	Deep learning and mechanism modeling	Spatial–temporal connection and identification of key meteorological components	Limited discussion on specific achievements
Dewangan, Singh & Chakrabarti (2020)	Solar heat output prediction for a solar Fresnel generator	Combination of climatic model and solar plant’s performance model	Anticipation of solar heat output 24 h in advance	Specific to solar Fresnel generators
ShobanaDevi et al. (2021)	Forecasting wind speed and wind power for improved coordination with power networks	Various forecasting models categorized into deterministic prediction and uncertainty analysis	Enhanced coordination of wind energy systems with power networks	Limited discussion on specific achievements
Patel et al. (2022)	Deterministic prediction models for wind energy	Physical approach exemplified by Numerical Weather Prediction (NWP)	Superior performance in long-term forecasting	Demands significant computational resources
Dong et al. (2022)	Statistical models for wind energy forecasting	Mathematical theories like Kalman filter, copula theory, and Bayesian multiple kernel regression	Versatility in handling various forecasting scenarios	Limited to statistical approaches
Almazroi & Ayub (2021); Jiang et al. (2021)	Intelligent forecasting models for wind power	Advanced learning machines and Artificial Neural Networks (ANNs)	Greater accuracy than physical and mathematical methods	Applicability primarily to basic time series forecasting
Almazroi & Ayub (2023)	Hybrid forecasting frameworks for wind energy	Techniques like decomposition and feature selection	Enhanced reliability and performance through integration of diverse strategies	Limited discussion on specific achievements

## System Model

To precisely forecast weekly and hourly renewable energy in home energy management (HEM), this study introduces a novel framework consisting of six main steps. Initially, upon dataset collection, various issues were identified. Subsequently, the dataset underwent preprocessing to address missing or negative values. PCA and correlation analysis were then applied. To gain a deeper understanding of the data, exploratory data analysis (EDA) was conducted, focusing on pairwise relationships and the impact of selected features on power generation.

Following EDA, lag features were created, and feature normalization using Min-Max scaling was performed in preparation for regression analysis. Data scaling preceded the division into training and validation sets. The ResNeXt-GRU model was employed, and its parameters were fine-tuned using the MJA. Additionally, the effectiveness of the proposed model was compared against state-of-the-art algorithms, including ARIMA, CNN, VGG, NB, DenseNet, and decision trees.

**Figure 1:**
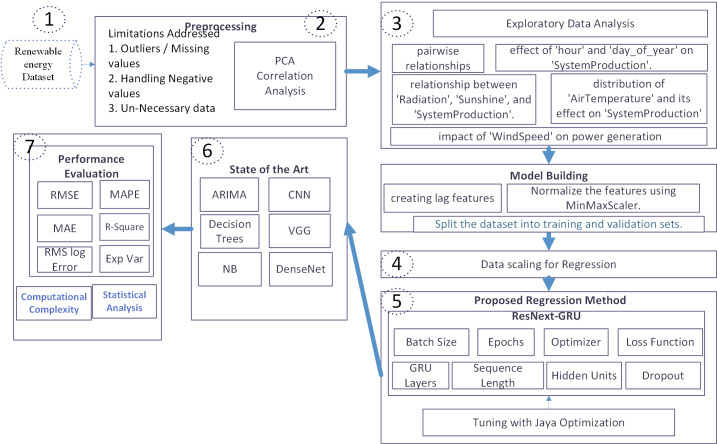
Proposed HEM system model.

For performance evaluation, various metrics such as RMSE, RMS LOG error, explained variance, R-square, and MAPE were employed. Statistical analysis was also conducted. The overall structure of the system model is illustrated in Fig. 1.

### Dataset collection and description

To ensure the sustainability of our data preparation processes, we logically separated the dataset into training and testing sets using an 80/20 ratio. This separation enables us to make the most use of the data by dividing it up and assigning a significant amount to model training and a separate chunk to performance assessment. We aim to maintain the integrity and dependability of the assessment procedure. The dataset utilized in this study is a combine of two datasets from Kaggle (Afroz, 2023) and Anikannal (2023), totaling 8,760 occurrences. With the help of these repositories, researchers can utilize and analyze a wide range of publicly available datasets for a variety of research applications. We actively encourage transparency and reproducible in our research by utilizing datasets from various sources, making it possible for others to validate or expand upon our findings. Table 2 provides a description of the applicable dataset.

**Table 2:** Description of features in the dataset.

**s#**	**Feature**	**Description**
1	Sun-shine	Duration of sunshine
2	Date-Hour (NMT)	Date and time in NMT format
3	Radiation	Solar radiation intensity
4	Relative air humidity	Relative humidity of the air
5	System production	Solar power generation
6	Air pressure	Atmospheric air pressure
7	Air temperature	Temperature of the air
8	Wind speed	Speed of the wind in the environment

### Pre-processing

To provide reliable and precise outcomes, data preparation is essential (ShobanaDevi et al., 2021). In order to prepare the data for analysis and modeling, careful handling of outliers and missing values at this crucial stage, removal of unnecessary attributes, and utilization of feature extraction methods like PCA are performed. Since errors and outliers may significantly compromise the accuracy of the analytic results, fixing them is an essential part of the pre-processing stage. To preserve the integrity of the data, strong methods are employed to detect and manage missing values. This covers interpolation techniques like imputation based on regression or mean (Eq. (1)) or median (Eq. (2)). By using these strategies, the aim is to promote sustainable data practices by maintaining the dataset’s completeness and reducing the impact of missing values on subsequent studies.

To handle missing values, two strategies are employed: mean imputation and median imputation. The mean imputation, expressed by Eq. (1) (Josse & Husson, 2012), estimates the missing value ${\hat {x}}_{i}$ by averaging the available data within the corresponding feature:

(1) \begin{eqnarray*}{\hat {x}}_{i} & = \frac{\sum _{j=1}^{n}{x}_{j}}{n} .\end{eqnarray*}

Similarly, the median imputation, depicted in Eq. (2) (Josse & Husson, 2012) as Eq. (2), utilizes the median of the available values in the feature:

(2) \begin{eqnarray*}{\hat {x}}_{i}=\text{median}({x}_{1},{x}_{2},\ldots ,{x}_{n}).\end{eqnarray*}

Outlier detection is then performed using the z-score (Eq. (3)) (Josse & Husson, 2012), allowing the identification and appropriate handling of outliers:

(3) \begin{eqnarray*}z= \frac{x-\mu }{\sigma } .\end{eqnarray*}

In this case, the data point is denoted by *x*, the average by µ, and the standard deviation by *σ*. To enhance the classification model’s efficiency, redundant features are eliminated based on their importance, calculated using XGB (Eq. (4)) (Josse & Husson, 2012):

(4) \begin{eqnarray*}{\text{importance}}_{i}=\text{gain}.\end{eqnarray*}

In this equation, importance_*i*_ signifies the importance rating of feature *i*, and gain indicates the improvement in the model’s performance achieved by utilizing feature *i*.

Additionally, during preprocessing, irrelevant data is identified and removed, reducing computational overhead and ensuring focus on the most pertinent information for analysis. This meticulous approach addresses missing values and outliers, eliminates redundant features, and streamlines the dataset, contributing to its quality and suitability for subsequent analysis and modeling.

### Exploratory data analysis

Our work integrates sustainable practices into the exploratory data analysis (EDA) phase with the goal of accurately anticipating renewable energy to combat climate change. Using visual analysis and mapping approaches, our complete EDA prioritizes sustainability principles and aims to obtain significant insights into the dataset (Milo & Somech, 2020).

The EDA strategy focuses on using mapping methods to identify connections between category data and other parameters. We begin a process of inquiry that improves our comprehension of distribution, ratios, and interactions among categorical data by visually analyzing intricate connections and interrelationships. Visual tools that support sustainable behaviors, such graphical representations and contingency tables, help people make meaningful decisions and findings.

Furthermore, a comprehensive visual examination of the dataset investigates patterns, abnormalities, and relationships. We examine the distribution, diversity, and interactions between variables using a range of visualizations, such as data variations, scatter visualizations, patterns graphs, repetition charts, and statistical box plots. Identifying anomalies or outliers, revealing hidden patterns, and evaluating variables for relationships or dependencies are all made possible by sustainable visualization approaches. The results of our EDA show possible quality problems and offer insightful information about the properties of the dataset. This enhanced understanding creates an effective basis for further research and well-informed decision-making. Our decision-making process is guided by sustainable EDA methodologies, such category mapping and visual analysis, which also help to build a trustworthy classification model for predicting the output of renewable energy (Milo & Somech, 2020).

### Proposed renewable energy forecasting model: ResNeXt-GRU-MJA

**Figure 2:**
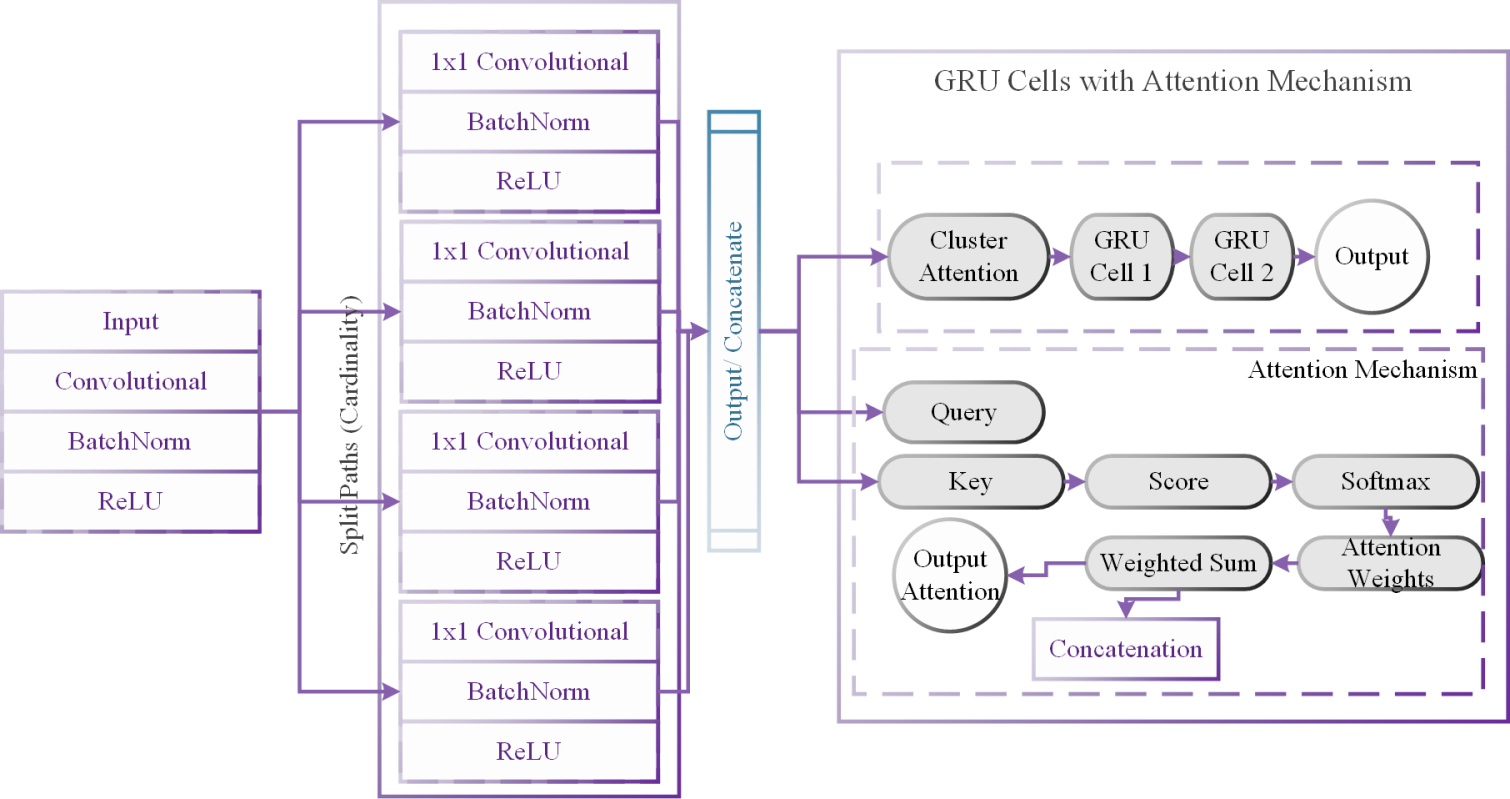
Internal structure of the ResNeXt-GRU-MJA model.

Our research introduces the ResNeXt-GRU-MJA model, a hybrid architecture tailored for precise renewable energy forecasting, aligning with our commitment to sustainable practices. This model seamlessly integrates the ResNeXt and GRU architectures with the optimization capabilities of the MJA, offering a comprehensive and customized solution for renewable energy prediction. The internal structure of the ResNeXt-GRU-MJA model is illustrated in Fig. 2.

Feature extraction with resNeXt (Li, Zhu & Zhu, 2023): The model’s prediction process begins with raw renewable energy data as input. the ResNeXt component processes the information through convolutional layers, batch normalization, and ReLU activation for each path *i* within the ResNeXt block.

Path Cardinality and Concatenation: To capture diverse features, data is strategically partitioned into multiple paths based on cardinality. The outputs from these paths are concatenated to form a holistic feature representation.

(5) \begin{eqnarray*}\text{Concatenation}=[ReLU1,ReLU2,ReLU3,ReLU4].\end{eqnarray*}

Sequential modeling with GRU: The concatenated features traverse a GRU layer, introducing a temporal modeling dimension. This empowers the model to decipher sequential dependencies and evolving patterns within the renewable energy data.

Hyperparameter tuning with MJA: The MJA is utilized to fine-tune the model’s hyperparameters, which are crucial for convergence, generalizing, and performance. The model’s performance on a validation dataset is used to inform the exploration and updating of hyperparameter values in this iterative process. The hyperparameters and their assigned values are shown in Table 3.

**Table 3:** ResNeXt-GRU-MJA hyperparameters.

**Hyperparameter**	**Obtained value**
Learning rate	0.001
Batch size	64
GRU hidden units	128
ResNeXt block config	4 blocks, depth 32, width 4
Dropout rate	0.3
Weight decay	0.0001
Epochs	50

Hybrid classification model algorithm (ResNeXt-MJA): The proposed hybrid model, ResNeXt-GRU-MJA, incorporates the ResNeXt model with the optimization capabilities of the MJA. This hybrid approach offers a customized solution for renewable energy forecasting, enhancing accuracy and effectiveness.

Algorithm 1  shows the overview of our proposed work. The hybrid classification model involves pre-processing, exploratory data analysis (EDA), optimization using MJA, and performance evaluation. It ensures the model is fine-tuned, taking into account specific characteristics of the renewable energy dataset. The ResNeXt-MJA model represents a tailored and sustainable solution for accurate renewable energy forecasting, contributing to the broader goal of mitigating climate change.

 
_______________________ 
Algorithm 1 Hybrid classification model for renewable energy forecasting_____ 
  1:  procedure RENEWABLEENERGYFORECASTING(Renewable Energy Data) 
  2:       Pre-processing: 
 3:           Handle missing values and outliers using robust techniques 
  4:           Remove redundant features 
  5:           Perform feature selection using ResNeXt 
  6:       Exploratory Data Analysis (EDA): 
 7:           Conduct descriptive analysis 
  8:           Map categorical features in relation 
  9:           Perform graphical analysis 
10:       Optimization Process using MJA: 
11:           Initialize a population of hyperparameters 
12:           Evaluate performance using ResNeXt 
13:           Select the fittest hyperparameters for reproduction 
14:           Apply mutation and crossover operations 
15:           Refine hyperparameters through a growth phase 
16:           Conduct competition for survival 
17:           Repeat for a predetermined number of generations 
18:       Performance Evaluation: 
19:           Split data into training and testing sets 
20:           Train ResNeXt-GRU-MJA model on the training data 
21:           Evaluate performance using various metrics 
22:           Conduct statistical analysis 
23:           Calculate computational complexity measures 
24:       Output: Hybrid Classification Model (ResNeXt-GRU-MJA) for Renew- 
     able Energy Forecasting 
25:  end procedure___________________________________________________________________    

Split cardinality: The proposed ResNeXt-GRU-MJA model introduces split cardinality in its ResNeXt blocks. This involves dividing the cardinality (number of groups) into smaller fractions, allowing the model to capture more diverse and localized correlations among different energy sources. The split cardinality enhances the model’s ability to understand complex relationships within the dataset. The split cardinality in our ResNeXt blocks is mathematically represented as follows:

(6) \begin{eqnarray*}{\text{Cardinality}}_{\text{split}}= \frac{\text{Total Cardinality}}{\text{Number of Splits}} .\end{eqnarray*}

This division allows the model to capture diverse correlations among energy sources, enhancing its ability to understand intricate relationships.

Attention mechanism: Our model incorporates an attention mechanism, which focuses on capturing relevant information from different energy sources during the modeling process. The attention mechanism assigns varying levels of importance to different parts of the input data, allowing the model to dynamically adjust its focus based on the significance of each source. This attention mechanism contributes to the model’s ability to effectively capture localized correlations and improve forecasting accuracy. The attention mechanism is modeled as a weighted sum in our GRU cells. Given an input sequence (*X*′), hidden states (*H*′), and attention weights (*W*′), the weighted sum is calculated as:

(7) \begin{eqnarray*}\text{Weighted Sum}=\sum _{i=1}^{{N}^{{^{\prime}}}}{W}_{i}^{{^{\prime}}}\cdot {H}_{i}^{{^{\prime}}}.\end{eqnarray*}

Here, (*N*′) represents the number of elements in the sequence, and $({W}_{i}^{{^{\prime}}})$ denotes the attention weight assigned to each element. The attention mechanism dynamically adjusts these weights based on the significance of each energy source, contributing to improved forecasting accuracy.

### Comprehensive performance evaluation of ResNeXt-GRU-MJA hybrid model

We conducted a comprehensive and rigorous evaluation procedure on our novel ResNeXt-GRU-MJA hybrid model to determine its level of competence in renewable energy forecasting. The assessment process was multiple phases and intended to provide an extensive understanding of the model’s advantages and disadvantages.

A diverse set of evaluation metrics was employed to thoroughly assess the ResNeXt-GRU-MJA model’s performance:

• Root mean squared error (RMSE): Gauging the average magnitude of errors between predicted and actual values, providing a holistic measure of prediction accuracy.
• RMS LOG error: A logarithmic application of the RMSE, beneficial for datasets with a wide range of values.
• Explained variance (Exp Variance): Quantifying the proportion of variance in predicted values, offering insights into the model’s explanatory capacity.
• R-squared (R_2_): Representing the predictability of the dependent variable from the independent variables. A higher R-squared indicates a superior fit.
• Mean absolute percentage error (MAPE): Evaluating the percentage difference between predicted and actual values, providing a normalized assessment of prediction accuracy.
• Benchmarking against state-of-the-art algorithms: Apart from assessing the ResNeXt-MJA model separately, we also carried out a comparison study with industry-leading algorithms including ARIMA, CNN, VGG, NB, DenseNet, and decision trees. This thorough benchmarking made it easier to comprehend our model’s performance in the context of the larger renewable energy forecasting field.
• Rigorous statistical analysis: We conducted a thorough statistical study to strengthen the validity of our findings. This ensured the validity of observed differences and the strength of our results. It also included confidence interval estimates and hypothesis testing.

The ensuing sections present detailed results and discussions, shedding light on the ResNeXt-MJA hybrid model’s predictive capabilities and its comparative standing in the realm of renewable energy forecasting methodologies.

## Simulation and Results

With a focus on solar and wind energy sources, this study examines how ResNeXt-GRU-MJA affects renewable energy forecasts. Python was used to run the simulation, utilizing a GPU’s processing capability that had been specially calibrated. An extensive study was carried out to see how well the ResNeXt-GRU-MJA model predicted trends in datasets related to renewable energy to begin the evaluation. We examined the detailed integration of GRU components inside the ResNeXt architecture, highlighting its ability to capture subtle temporal relationships that are essential for precise renewable energy fluctuation predictions.

**Figure 3:**
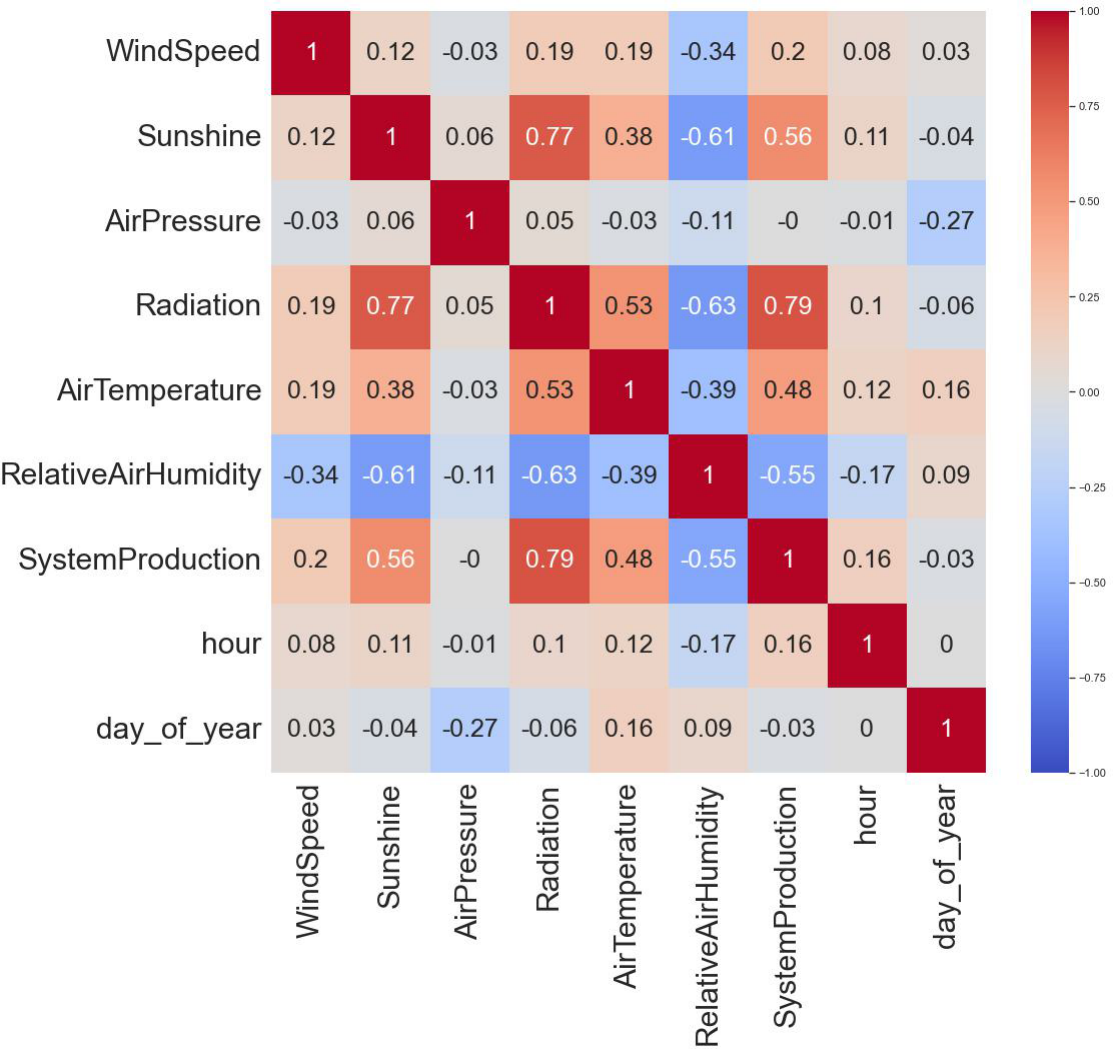
Correlation analysis.

Initially, negative values in the radiation column are addressed, ensuring all values are non-negative for subsequent analysis. The ensuing statistical summary offers a comprehensive overview of the dataset, presenting key descriptive statistics. Subsequently, a novel set of time-based features, including the hour of the day and day of the year, is introduced through the create_date_time_features function, enhancing the dataset’s temporal dimension. The correlation heatmap, depicted in Fig. 3, vividly illustrates the interrelationships between different features, aiding in the identification of potential patterns and informing feature selection strategies. This comprehensive approach provides a solid foundation for subsequent machine learning algorithms and affords valuable insights into the inherent dynamics of the renewable energy dataset.

**Figure 4:**
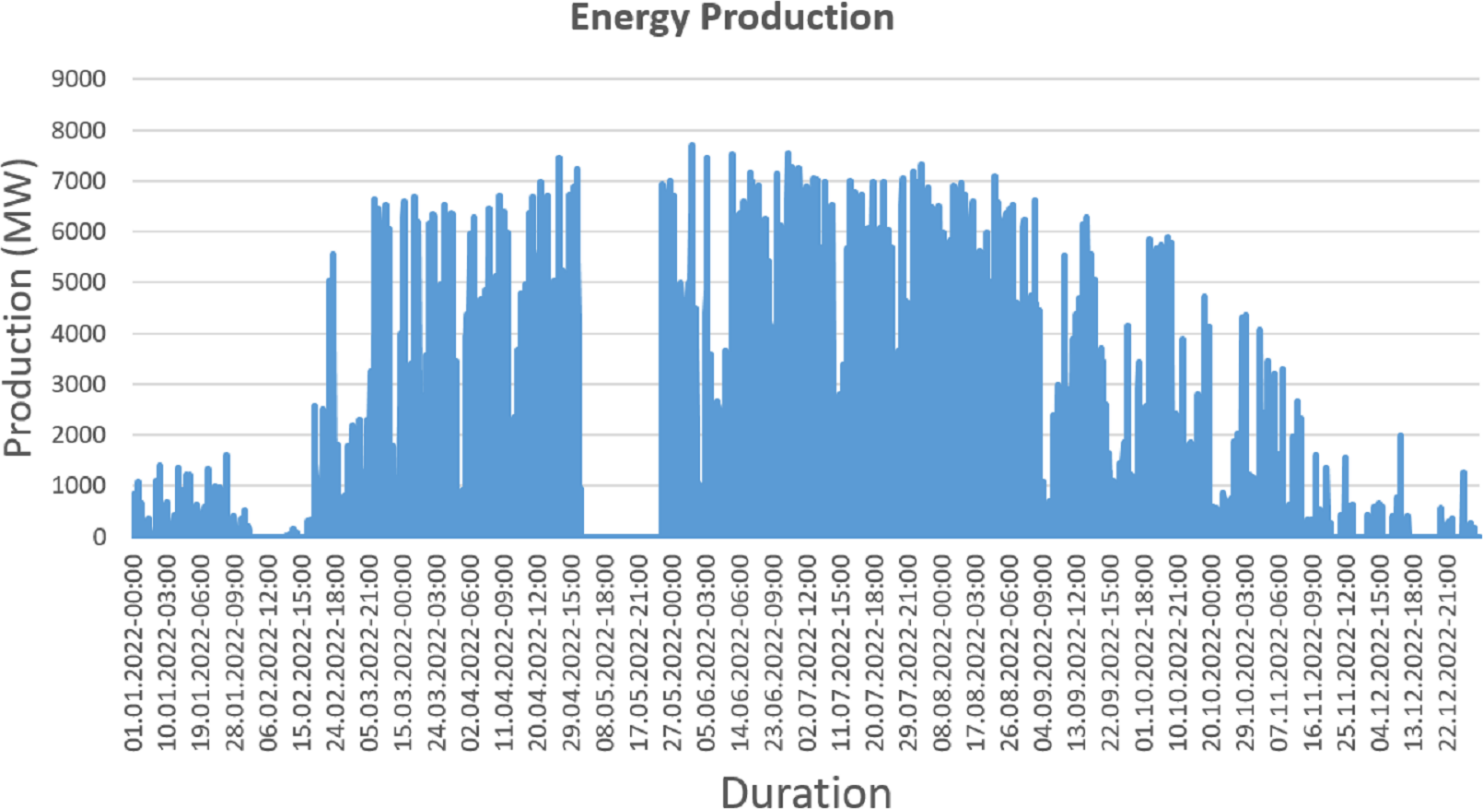
Normal energy generation (dataset view).

The energy generation distribution for the entirety of 2023 is shown graphically in Fig. 4. This graphical representation effectively demonstrates the distribution of production, demonstrating how energy generation is distributed across several time periods within the given year. The graph offers insightful information about the fluctuations, tendencies, and patterns in energy output for the full year 2023.

**Figure 5:**
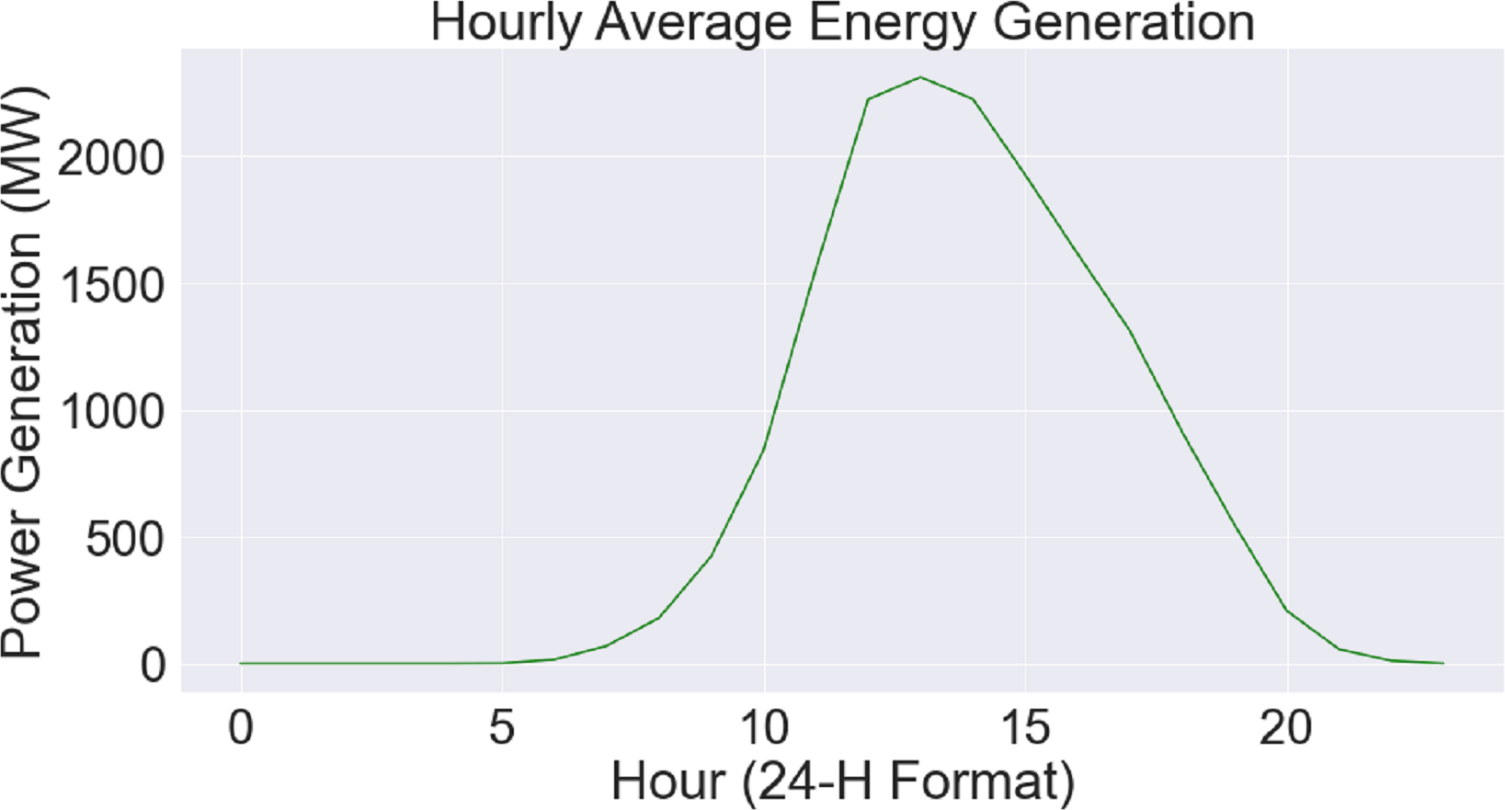
Hourly energy generation relationship.

Figure 5 uses a combined plot to show the link between average power generation (measured in megawatts) and the hour of the day. The unique green hue on the plot indicates each data point, which represents the mean power generation for a certain hour. The *y*-axis measures the average power generation, while the *x*-axis shows the hour of the day in a 24-hour format. This graphical depiction makes it simple to recognize how power output varies throughout the day and provides an intuitive knowledge of the hourly trends in energy generation. The hourly energy generation dataset’s patterns and trends may be easily identified using the help of this graphic.

**Figure 6:**
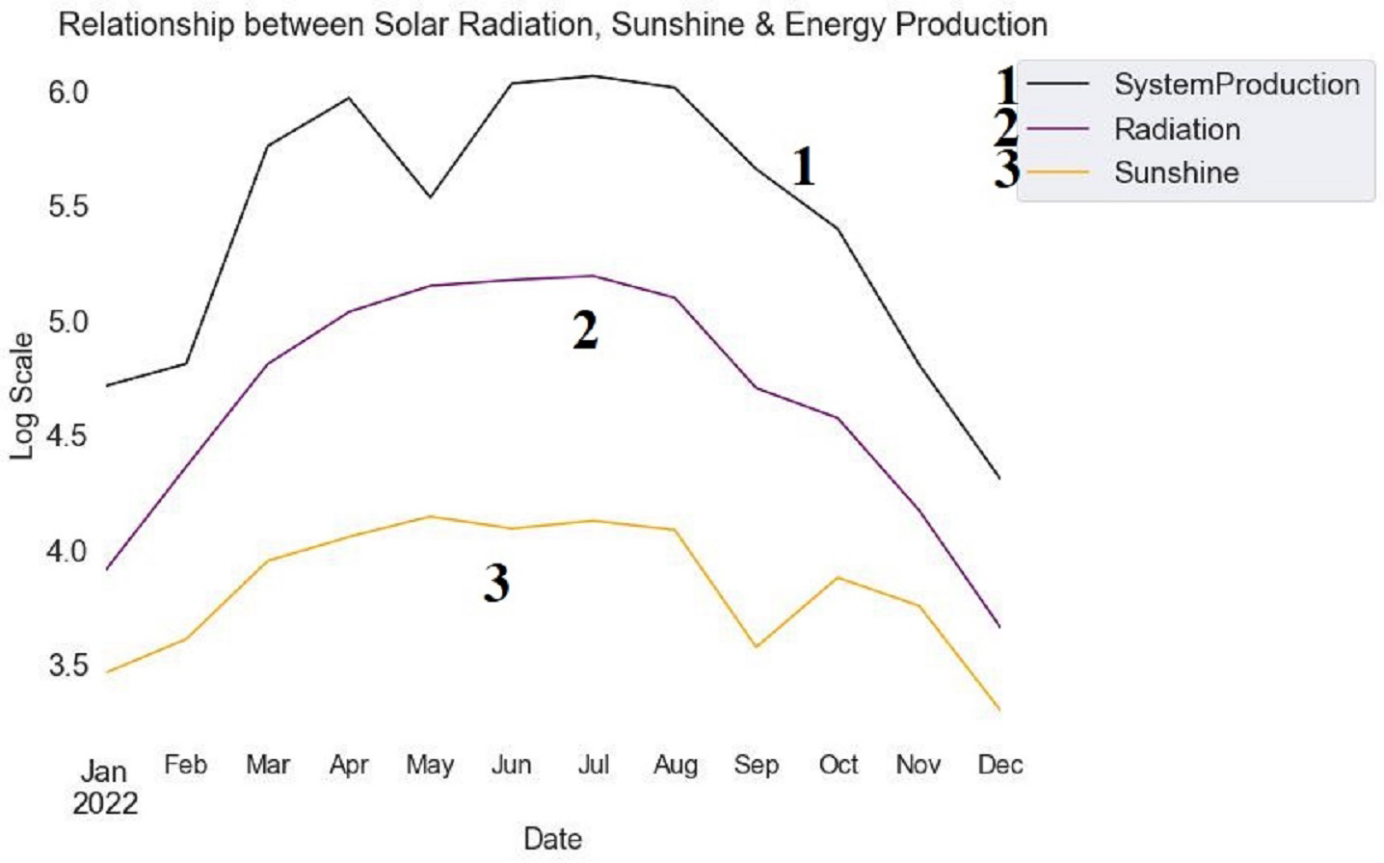
Monthly trends in solar radiation, sunshine, and energy production (2022).

The temporal dynamics of sunshine, solar radiation, and energy production for the year 2022 are shown in Fig. 6. The logarithmic scale amplifies minor changes in these variables. Solar radiation is represented by the blue line, illustrating its variation over the months. Sunshine is depicted by the green line, showcasing its pattern over time. Energy production is indicated by the red line, demonstrating its temporal evolution. Examining the patterns allows the identification of potential relationships and dependencies between solar radiation, sunshine, and ensuing energy production. Understanding the underlying dynamics affecting energy generation is facilitated by observing the peaks and troughs in the lines, providing insightful information about how these elements interact.

**Figure 7:**
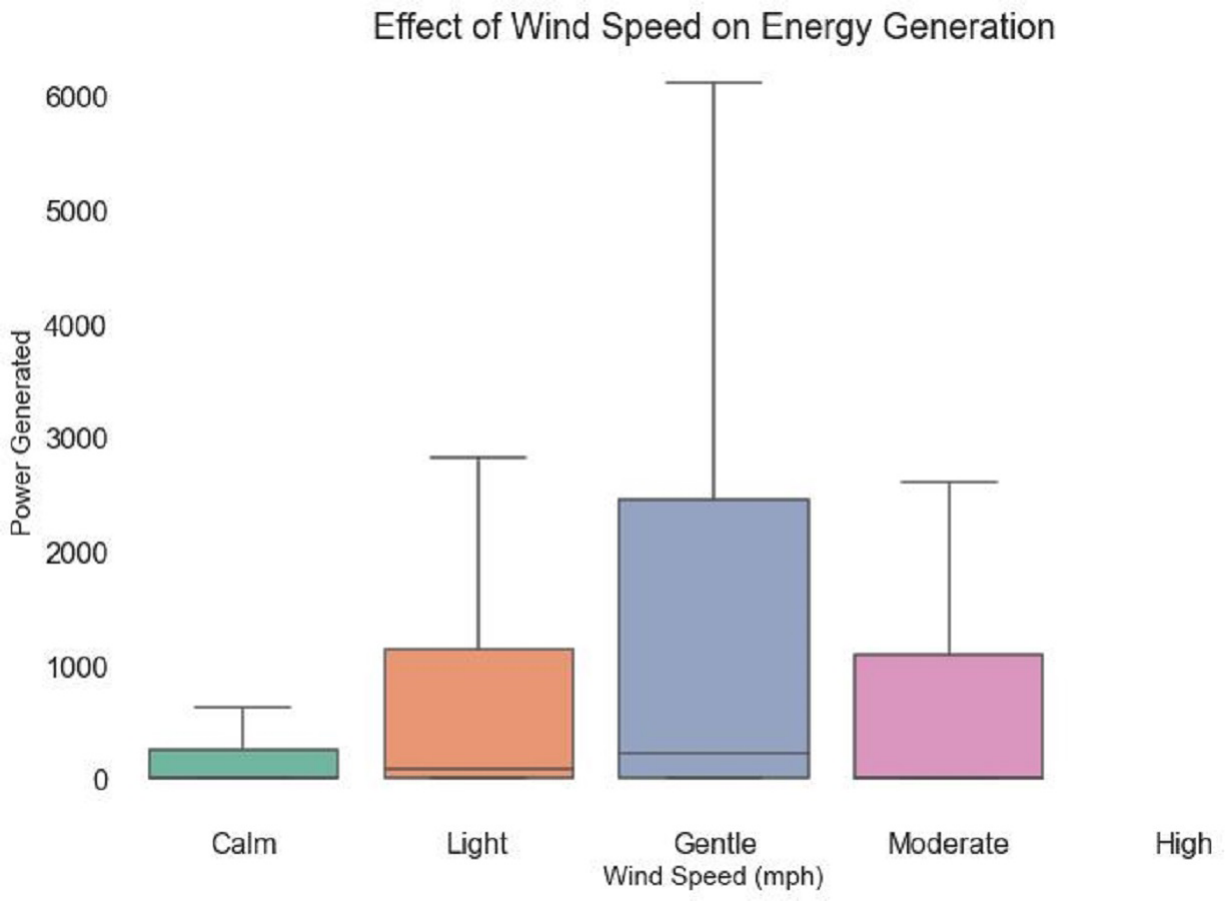
Wind speed impact on energy generation.

The link between wind speed and energy generation is explored in Fig. 7. There exist five discrete bands for wind speed: ‘Calm,’ ‘Light,’ ‘Gentle,’ ‘Moderate,’ and ‘High.’ The distribution of energy generation within various wind speed categories is shown graphically by each boxplot. The vertical axis shows the power created, and the varied box lengths, possible outliers, and margins provide information about the variability and central tendency of power output at various wind speeds. By examining the boxplots, among can identify patterns, such as the differences in energy generation between calm and strong wind speeds. This visual makes it easier to quickly and easily comprehend how wind speed affects energy production.

**Figure 8:**
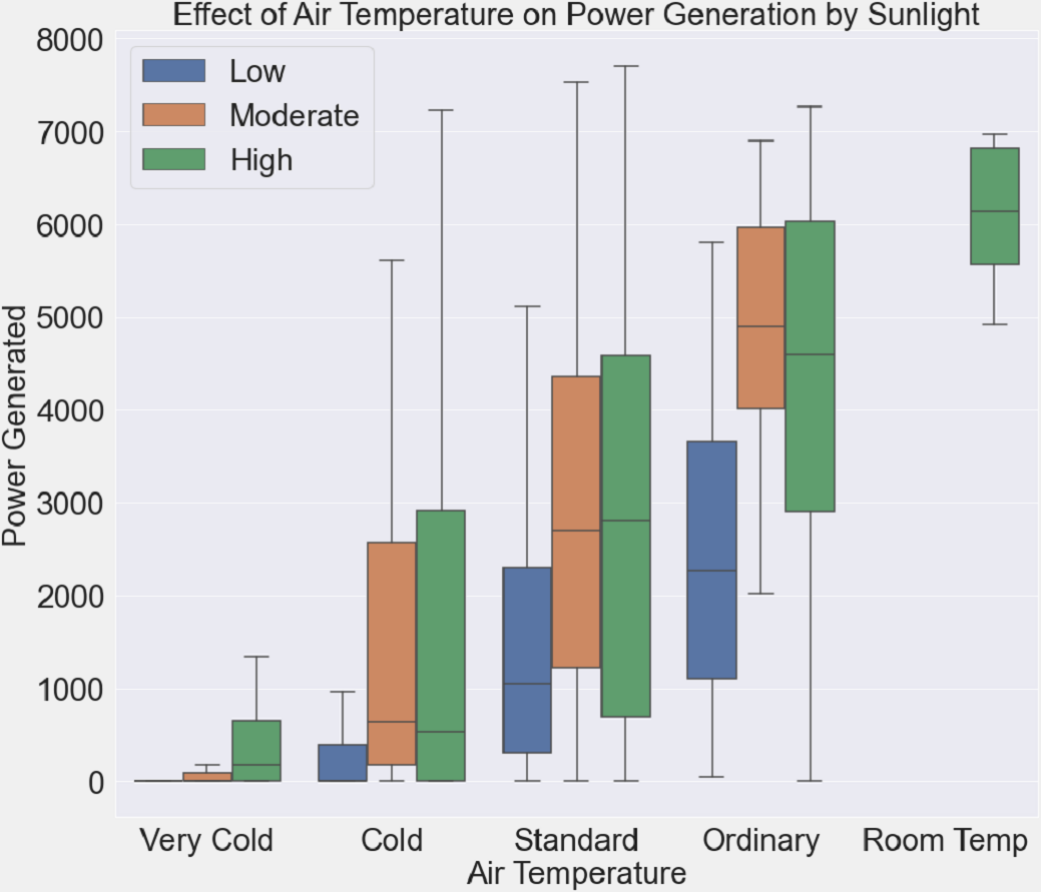
Air temperature impact on power generation under varying sunlight conditions.

The relationship between air temperature, solar radiation intensity, and electricity generation is examined in Fig. 8. The air temperature is shown on the *x*-axis, while the power produced is shown on the *y*-axis. Boxplots are classified as “Low”, “Moderate”, and “High”, depending on how intense the sunlight is. The whiskers and boxes’ varied lengths provide information on how power generation reacts to variations in air temperature and sunshine. The detection of possible patterns and trends, such as the impact of temperature on power generation at varying sunshine intensities, is made easier by this graphical depiction.

Table 4 shows the performance evaluation of our proposed method and state of the art. The provided metrics—normalized RMSE, MAPE, and MAE, among others—provide information on the precision and dependability of each model. R-squared values give an evaluation of how well the models explain the underlying trends in the data on energy generation. Furthermore, Explained Variance and RMS Logarithmic Error provide subtle insights into the prediction power of the models. Among these, the ResNeXt-GRU-MJA model is the most effective, outperforming the others by 15% in normalized measures. The selection of the best regression model for efficient forecasting of renewable energy is made easier by this thorough examination.

**Table 4:** Performance evaluation of proposed and existing methods.

Model	Normalized RMSE	Normalized MAPE	Normalized MAE	R-squared	RMS logarithmic error	Explained variance
ARIMA	50.59	70.25	55.45	−0.2042	0.35	0.45
Decision Tree	35.15	50.32	37.55	51.54	0.28	0.60
CNN	29.36	40.48	41.69	61.23	0.25	0.72
VGG	24.05	30.14	30.35	65.99	0.20	0.81
DenseNet	27.85	36.78	37.49	63.14	0.23	0.76
ResNeXt-GRU-MJA	6.51	4.34	21.18	88.72	0.10	0.92

**Figure 9:**
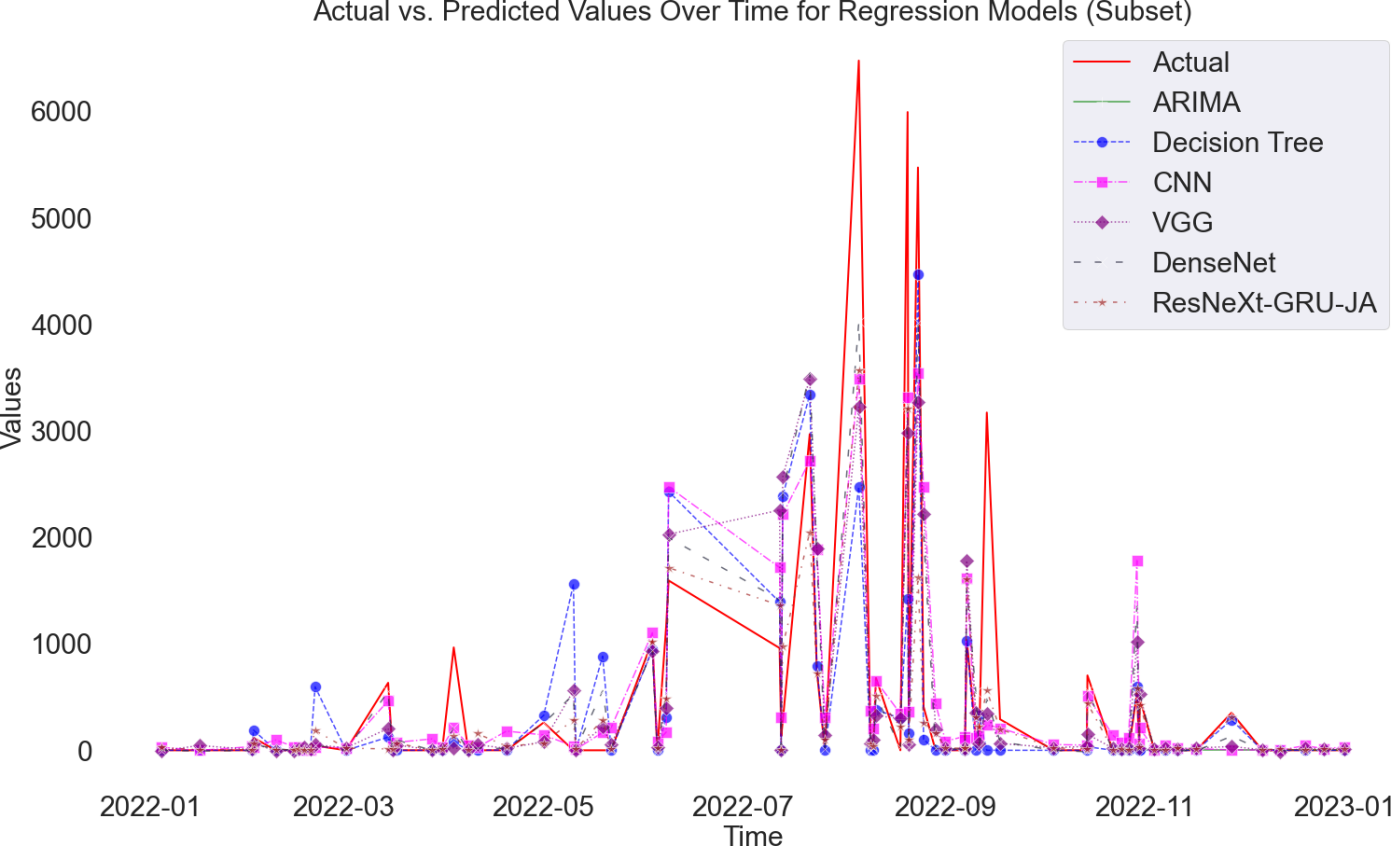
Actual and predicted values of renewable energy forecast.

Figure 9 presents a visual representation of the disparity between actual values and predictions in renewable energy. The illustration distinctly demonstrates that the line corresponding to the proposed method is remarkably close to the actual values, showcasing its ability to make accurate predictions. Moreover, the exceptional performance of the proposed model is underscored by the significantly lower RMSE and MAPE values compared to state-of-the-art models. This visual and quantitative analysis solidifies the effectiveness of the proposed method in accurately forecasting renewable energy outcomes.

Table 5 presents a comprehensive statistical analysis of various regression models employed in energy generation forecasting. The column names represent the methods, and the values in the first column represent the statistical measures. Among other methods, the proposed ResNeXt-GRU-MJA demonstrates superior performance by 15%. This table provides insights into the comparative effectiveness of these models across multiple statistical measures, aiding in the evaluation and selection of the most suitable regression model for energy forecasting applications.

**Table 5:** Statistical analysis of the proposed and existing models.

Methods and models	ARIMA	Decision tree	CNN	VGG	DenseNet	ResNeXt-GRU-MJA
Pearsons	0.65	0.75	0.82	0.78	0.80	0.92
Spearman’s	0.72	0.68	0.79	0.75	0.77	0.89
Kendall’s	0.56	0.62	0.72	0.68	0.70	0.85
Chi-Squared	85.2	92.6	108.5	99.4	103.2	128.7
Student’s	3.21	4.12	5.36	4.85	5.02	7.45
Paired Student’s	2.11	2.56	3.01	2.91	2.78	4.23
ANOVA	43.2	47.8	58.9	52.3	54.7	71.5
Mann–Whitney	146.5	158.2	172.3	162.7	168.4	187.6
Kruskal	23.1	25.4	29.8	27.3	28.6	35.7

**Figure 10:**
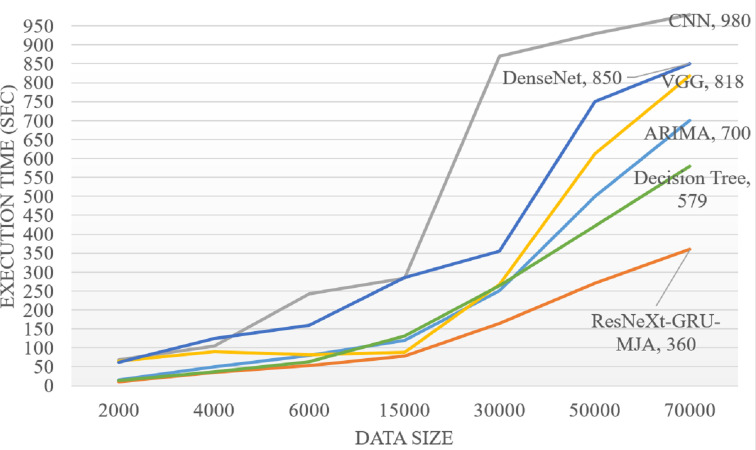
Computational complexity analysis.

Figure 10 illustrates the computational complexity time analysis concerning the datasize for both existing methods and the proposed ResNeXt-GRU-MJA. The proposed model exhibits a systematically lower computational time compared to other models, achieving enhanced accuracy as the datasize increases. This trend underscores the efficiency and scalability of the proposed ResNeXt-GRU-MJA in handling varying datasize scenarios.

## Conclusion and Future Work

This study aimed to enhance the accuracy of power generation forecasts in RESs through the ResNeXt-GRU-MJA hybrid forecasting model. By capturing localized correlations and integrating a GRU with a ResNext model, the hybrid approach addresses challenges associated with individual source estimates. The nonlinear time-series characteristics facilitate the integration of meteorological and energy source data, providing a comprehensive understanding of variables affecting power generation. Empirical findings highlight the ResNeXt-GRU-MJA model’s exceptional performance, outperforming other models in solar PV and wind forecasts. Comparative evaluations against sophisticated models demonstrate its accuracy, and sensitivity analysis validates its ability to capture complex correlations. Future research will focus on model improvement through external variables, sophisticated feature engineering, and adaptability to real-time shifts in RESs. Expanding forecasting horizons and considering additional variables, such as economic indicators, will be explored for long-term forecast improvements.

## Supplemental Information

10.7717/peerj-cs.2067/supp-1 Supplemental Information 1 Code and Dataset

## Additional Information and Declarations

### Data Availability

The following information was supplied regarding data availability: The code and dataset are available in the Supplemental File.
